# Minimally-Myelosuppressive Asparaginase-Containing Induction Regimen for Treatment of a Jehovah’s Witness with mutant *IDH1/NPM1/NRAS* Acute Myeloid Leukemia

**DOI:** 10.3390/ph9010012

**Published:** 2016-03-10

**Authors:** Ashkan Emadi, Najeebah A. Bade, Brandi Stevenson, Zeba Singh

**Affiliations:** 1 School of Medicine, Marlene & Stewart Greenebaum Cancer Center, University of Maryland, 22 South Greene Street, Room N9E24, Baltimore, MD 21201, USA; nabade@gmail.com (N.A.B.); bstevenson@umm.edu (B.S.); 2 Department of Pathology, School of Medicine, University of Maryland, Baltimore, MD 21201, USA; zsingh@umm.edu

**Keywords:** acute myeloid leukemia (AML), isocitrate dehydrogenase (IDH), asparaginase

## Abstract

Treatment of patients with acute myeloid leukemia (AML) who do not wish to accept blood product transfusion, including Jehovah’s Witnesses, is extremely challenging. The use of conventional chemotherapy for induction of complete remission (CR) results in profound anemia and thrombocytopenia requiring frequent transfusions of blood products, without which such treatment will be life-threatening. Finding a well tolerable, minimally myelosuppressive induction regimen for such patients with AML is a clear example of area of unmet medical need. Here, we report a successful treatment of a 52-year-old Jehovah’s Witness with newly diagnosed AML with peg-asparaginase, vincristine and methylprednisolone. The AML was characterized with normal karyotype, and mutations in isocitrate dehydrogenase 1 (IDH1-Arg132Ser), nucleophosmin 1 (NPM1-Trp289Cysfs*12) and neuroblastoma RAS viral oncogene homolog (NRAS-G1y12Va1). After one 28-day cycle of treatment, the patient achieved complete remission with incomplete count recovery (CRi) and after the second cycle, he achieved CR with full blood count recovery. The patient has never received any blood products. Notwithstanding that myeloperoxidase-induced oxidative degradation of vincristine results in its lack of activity as monotherapy in AML, its combination with corticosteroid and asparaginase has resulted in a robust remission in this patient. Diminished steroid clearance by asparaginase activity as well as reduction in serum glutamine level induced by glutaminase enzymatic activity of asparaginase may have contributed to effective killing of the myeloblasts that carry *IDH1*/*NPM1*/*NRAS* mutations. In conclusion, asparaginase-containing regimens, which are approved for treatment of acute lymphoblastic leukemia (ALL) but not AML, can be used to treat patients with AML who do not accept blood transfusion.

## 1. Introduction

Treatment of individuals who do not accept transfusion of blood products, including Jehovah’s Witnesses, with acute myeloid leukemia (AML) is exceptionally challenging [1,2,3]. Conventional remission induction regimens containing cytotoxic agents such as cytarabine and anthracyclines often are not offered to these patients, because chemotherapy results in profound myelosuppression requiring frequent red blood cell and platelet transfusions without which severe anemia and thrombocytopenia are life-threatening [4,5,6]. On the other hand, without induction treatment, these patients rapidly will succumb to their leukemia.

Asparaginase products, which are approved for treatment of acute lymphoblastic leukemia (ALL) [7,8,9], but not AML, do not suppress the bone marrow and can provide a treatment option for patients with AML who do not wish to receive blood transfusion [10,11]. In this article, we report and discuss a successful treatment of a Jehovah’s Witness with newly diagnosed AML with an asparaginase-containing regimen.

### Case Report

**Figure 1:**
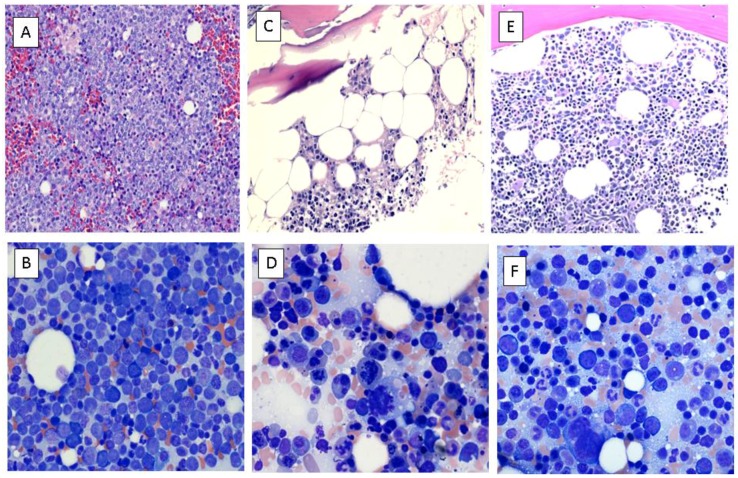
Bone marrow aspirate, biopsy and clot section smears. (**A**) Clot section at diagnosis (Hematoxylin and Eosin [H&E] stain ×20) showing large hypercellular marrow fragments (cellularity >90%) with predominantly blasts (70%) admixed with maturing erythroid precursors and lymphocytes. Megakaryocytes are identified, but decreased in proportion to the cellularity. Maturing granulopoiesis is markedly reduced; (**B**) Bone marrow aspirate at diagnosis (Wright stain ×40) showing myeloid:erythroid (M:E) ratio of 3:1, markedly reduced and left-shifted granulopoiesis, blasts comprising 52% of the cellularity; (**C**) Bone marrow core biopsy on day 28 (H&E stain ×20) showing maturing erythroid precursors as the majority of the cells, lymphocytes, and a few megakaryocytes with decreased granulopoiesis and *no overt increase in blasts*; (**D**) Bone marrow aspirate on day 28 (Wright stain ×40) showing decreased M:E ratio (0.5:1), most of the cells being of erythroid lineage and lymphocytes with a few maturing granulocytes and mildly dysplastic megakaryocytes related to chemotherapy and *no increase in blast cells*; (**E**) Bone marrow core biopsy on day 81 (H&E stain ×20) showing normocellular (50%–60%) marrow for the age of the patient, maturing granulopoiesis and erythropoiesis with an erythroid predominance, adequate megakaryocytes focally clustered with occasional small forms, no lymphoid aggregates, and *no myeloblasts*; (**F**) Bone marrow aspirate on day 81 (Wright stain x40) showing M:E ratio of 0.8:1, full spectrum maturation in the granulocytic and erythroid series, megakaryocytes with normal morphology, no increase in lymphocytes, plasma cells, or presence of any other abnormal cell population including *no increase in blasts*.

A 52-year-old Jehovah’s Witness presented with a few weeks of fatigue, weight loss, and pancytopenia; white-cell count (WBC) 7.3 × 10^9^/L with 57% peripheral blast, hemoglobin 6.2 g/dL, platelet 64 × 10^9^/L. Bone marrow aspiration and biopsy confirmed the diagnosis of AML (Figure 1A,B).

Immunophenotypic studies by flow cytometry on the bone marrow specimen showed 60%–70% blasts expressing CD117 (heterogeneous), CD33, CD13, CD15 (partial), CD11b (heterogeneous), CD11c, CD38, CD64 (partial), CD71 (partial), and myeloperoxidase. The blasts were negative for CD34, HLA-DR, CD14, CD16, CD56, TdT, and all T- and B-lymphoid antigens. Chromosome analysis revealed 46XY in 20 metaphase cells. Mutational analysis of the DNA from bone marrow blasts revealed mutations in isocitrate dehydrogenase 1 (*IDH1*-Arg132Ser, nucleotide change: c.394C > A, mutation level: 50%), nucleophosmin 1 (*NPM1*-Trp289Cysfs*12, nucleotide change: c.860_863dupTCTG, mutation level: 41%) and neuroblastoma RAS viral oncogene homolog (*NRAS*-G1y12Va1, nucleotide change: 0.35G > T, mutation level: 27%). Due to religious belief, the patient did not accept transfusion of red blood cells, platelets or other blood products but wished to proceed with treatment. After a comprehensive discussion of risks and benefits with the patient and his family, a minimally myelosuppressive treatment with peg-asparaginase (2500 units/m^2^ intravenously on day 8), vincristine (2 mg intravenously on days 1, 6, 11, 16, 21, 26) and methylprednisolone (20 mg/m^2^ twice daily on days 1–28) was initiated [11].

**Figure 2:**
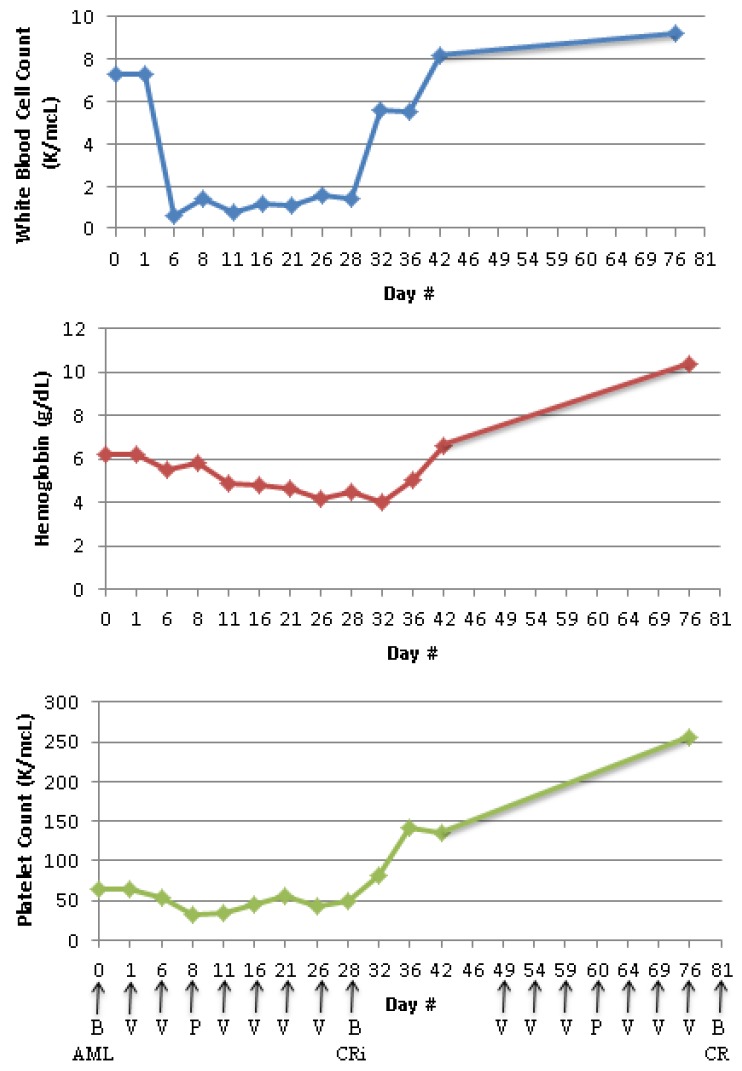
White blood cell, hemoglobin, and platelet counts from diagnosis throughout two cycles of chemotherapy regimens; Arrows indicate the days of bone marrow biopsy (B), receiving vincristine (V), or pegasparaginase (P).

He tolerated the chemotherapy regimen well without developing any major adverse events. Even though, his hemoglobin and platelet counts remained low (Figure 2), he did not experience any symptoms indicating cardio-respiratory compromise or any bleeding, and did not receive any blood product transfusion.

Twenty eight days after initiation of the chemotherapeutic agents, a bone marrow aspiration and biopsy was repeated which showed cellular marrow with no morphological or immunophenotypic evidence of AML (Figure 1C,D) with a decrease in blasts from 55% by flow cytometry to <1%. On the same day, complete blood count (CBC) showed WBC 1.4 × 10^9^/L with 53% granulocyte, no blasts, hemoglobin 4.5 g/dL, and platelet 50 × 10^9^/L (Figure 2), confirming the achievement of complete remission with incomplete count recovery (CRi). Due to logistical issues including insurance coverage, serum glutamine and 2-hydroxyglutarate (2-HG) levels could not be measured.

One week later, the patient developed edema in the right upper extremity where had a peripherally inserted central catheter (PICC) as well as left lower extremity edema. The duplex ultrasound revealed acute thrombus in the right distal subclavian vein, right axillary vein and right brachial vein as well as acute, occlusive deep venous thrombosis (DVT) of the paired left peroneal veins (isolated, axial calf DVT) without extension proximally into the left popliteal vein or the more-proximal deep veins of the left leg. Since at this point patient had adequate platelet recovery to 142 × 10^9^/L, he was treated with full dose low molecular weight heparin. His symptoms had resolved in a few days. 

Forty nine days after diagnosis and initiation of the first cycle of induction treatment, he began the second cycle of the same chemotherapy regimen with pegasparaginase, vincristine, and prednisone (50% dose reduced) as an outpatient. Patient remained on anticoagulation and tolerated the chemotherapy well except for development of Grade 2 vincristine-induced peripheral neuropathy. The CBC upon completion of the second cycle showed WBC 9.2 × 10^9^/L with 54% granulocytes, no blasts, hemoglobin 10.4 g/dL, and platelets 255 × 10^9^/L (Figure 2). His bone marrow aspiration and biopsy revealed normocellular bone marrow for age (cellularity 50%–60%) with full-spectrum trilineage hematopoiesis, and no morphological or immunophenotypic evidence of involvement by AML, confirming the achievement of CR (Figure 1E,F). The re-analysis of the bone marrow cells for the myeloid mutation panel revealed no somatic mutations or disease specific alterations, including the original *IDH1*, *NPM1*, and *NRAS* indicating achievement of molecular remission. His performance status and organ functions have remained normal.

## 2. Discussion

Both *E. coli* and *Erwinia*-derived asparaginase products including long acting pegasparaginase (polyethylene glycol L-asparaginase) possess a dual asparaginase and glutaminase enzymatic activity that diminishes both serum asparagine and glutamine levels by deamidating them to aspartate and glutamate, respectively [12]. It is believed that the cytotoxicity of asparaginase against lymphoblasts is due to auxotrophic nature of these cells for asparagine; however, the therapeutic value of asparaginases against myeloblasts is reported to be due to glutamine depletion resulting in disruption of protein synthesis downstream of mammalian target of rapamycin (mTOR) causing strong apoptotic and autophagic responses [13,14,15,16].

Mutations in *IDH1* and *IDH2* have been reported in approximately 20% of *de novo* AML [17,18,19,20,21], and their presence is an unfavorable prognostic factor according to most studies [22,23,24,25,26]. It has been suggested that *IDH* mutations confer adverse prognostic effect in patients with AML whose myeloblasts lack *NPM1* mutation [26,27]. Wild-type IDHs are NADP-dependent enzymes that catalyze the oxidative decarboxylation of isocitrate to α-ketoglutarate (α-KG), with production of NADPH [28]. Altered amino acids in mutant IDH are located in the catalytic pocket of the enzymes resulting in converting α-KG to 2-hydroxyglutarate (2-HG) with the consumption of NADPH. Heterozygous mutations resulting in a single amino acid change at arginine 132 (R132H, R132C, R132G, R132S) of IDH1 and arginine 140 (R140Q, R140W) or arginine 172 (R172K, R172G) of IDH2 have been reported [18,20]. By ^13^C and isotope-labeling experiments, it has been shown that the primary source for α-KG in AML cells with *IDH* mutation is glutamine [18]. Subsequently, it has been demonstrated that *in vitro* interruption of glutamine metabolism preferentially slows the growth of primary AML cells with mutant *IDH versus* wild type *IDH* [29,30]. Clinical studies aiming at the evaluation of safety and efficacy of asparaginase products in patients with AML with or without *IDH* mutations are ongoing (NCT02283190 and NCT01810705). 

The major adverse events of asparaginase products, reported from clinical trials involving patients with ALL, include anaphylaxis and serious allergic reactions, thrombosis, pancreatitis, glucose intolerance, coagulopathy, and hepatotoxicity. Interestingly, myelosuppression does not occur frequently and seriously after asparaginase administration. Also the myelosuppressive effect of vincristine compared to other conventional chemotherapeutic agents is minimal.

The fact that asparaginase and vincristine are minimally myelosuppressive is important for patients such as Jehovah’s Witnesses that do not accept blood product transfusion; as it is well known that the use of conventional chemotherapy for remission induction in AML results in life-threatening anemia and thrombocytopenia, requiring blood product transfusions with an average of 10.8 and 8.5 units of red blood cell and platelet transfusions, respectively [31]. We combined asparaginase and steroid with vincristine to increase their anti-leukemic effect, while maintaining the minimal myelosuppressive property of the regimen. Vincristine, a cell cycle-specific vinca alkaloid which induces metaphase arrest, as a single agent is not active against myeloblasts in AML [32]. Overexpression of multi-drug resistance proteins and myeloperoxidase-induced oxidation and degradation of vincristine are the main causes of inherent resistance of AML cells to vincristine as monotherapy [33,34]. While a synergistic or additive anti-leukemic activity between vincristine and asparaginase is not well known; decreased clearance of steroid with sustained asparaginase activity combined with prolonged asparaginase half-life due to immunomodulatory effect of steroid on anti-asparaginase antibody suggest synergism between asparaginase and steroid products [35,36].

## 3. Conclusion

A minimally myelosuppressive regimen including pegasparaginase, vincristine and steroid was used to induce durable complete remission in a patient with mutant *IDH1/NPM1/NRAS* AML who did not want to receive blood product transfusions. Moving forward, we plan to continue using non-myelosuppressive mono- or combination-therapies as consolidation regimens for this patient. We also plan to prospectively test the effect of this combination for similar patients with prospective measurement of serum biomarkers.

## References

[1] Kerridge I., Lowe M., Seldon M., Enno A., Deveridge S. (1997). Clinical and ethical issues in the treatment of a jehovah’s witness with acute myeloblastic leukemia. Arch. Intern. Med..

[2] Cullis J.O., Duncombe A.S., Dudley J.M., Lumley H.S., Apperley J.F., Smith A.G. (1998). Acute leukaemia in jehovah’s witnesses. Br. J. Haematol..

[3] Brown N.M., Keck G., Ford P.A. (2008). Acute myeloid leukemia in jehovah witnesses. Leuk. Lymphoma.

[4] Emadi A., Karp J.E. (2014). The state of the union on treatment of acute myeloid leukemia. Leuk. Lymphoma.

[5] Emadi A., Karp J.E. (2012). The clinically relevant pharmacogenomic changes in acute myelogenous leukemia. Pharmacogenomics.

[6] Bhatnagar B., Duong V.H., Gourdin T.S., Tidwell M.L., Chen C., Ning Y., Emadi A., Sausville E.A., Baer M.R. (2014). Ten-day decitabine as initial therapy for newly diagnosed patients with acute myeloid leukemia unfit for intensive chemotherapy. Leuk. Lymphoma.

[7] F.D.A. Label for asparaginase. http://www.accessdata.fda.gov/drugsatfda_docs/label/2013/101063s5169lbl.pdf.

[8] F.D.A. Label for erwinaze. http://www.accessdata.fda.gov/drugsatfda_docs/label/2011/125359lbl.pdf.

[9] F.D.A. Label for oncaspar. http://www.accessdata.fda.gov/drugsatfda_docs/label/2014/103411s5180lbl.pdf.

[10] Emadi A., Zokaee H., Sausville E.A. (2014). Asparaginase in the treatment of non-all hematologic malignancies. Cancer Chemother. Pharmacol..

[11] Fujita H., Iguchi M., Tachibana T., Takemura S., Taguchi J., Tanaka M., Maruta A., Ishigatsubo Y. (2006). Remission induction treatment for 6 patients of jehova’s witnesses with de novo acute leukemia. Blood.

[12] Avramis V.I. (2011). Asparaginases: A successful class of drugs against leukemias and lymphomas. J. Pediatr. Hematol. Oncol..

[13] Willems L., Jacque N., Jacquel A., Neveux N., Maciel T.T., Lambert M., Schmitt A., Poulain L., Green A.S., Uzunov M. (2013). Inhibiting glutamine uptake represents an attractive new strategy for treating acute myeloid leukemia. Blood.

[14] Samudio I., Konopleva M. (2013). Asparaginase unveils glutamine-addicted aml. Blood.

[15] Jacque N., Ronchetti A.M., Larrue C., Meunier G., Birsen R., Willems L., Saland E., Decroocq J., Thiago T.T., Lambert M. (2015). Targeting glutaminolysis has antileukemic activity in acute myeloid leukemia and synergizes with bcl-2 inhibition. Blood.

[16] Emadi A. (2015). Exploiting aml vulnerability: Glutamine dependency. Blood.

[17] Mardis E.R., Ding L., Dooling D.J., Larson D.E., McLellan M.D., Chen K., Koboldt D.C., Fulton R.S., Delehaunty K.D., McGrath S.D. (2009). Recurring mutations found by sequencing an acute myeloid leukemia genome. New Engl. J. Med..

[18] Dang L., White D.W., Gross S., Bennett B.D., Bittinger M.A., Driggers E.M., Fantin V.R., Jang H.G., Jin S., Keenan M.C. (2009). Cancer-associated idh1 mutations produce 2-hydroxyglutarate. Nature.

[19] Chou W.C., Hou H.A., Chen C.Y., Tang J.L., Yao M., Tsay W., Ko B.S., Wu S.J., Huang S.Y., Hsu S.C. (2010). Distinct clinical and biologic characteristics in adult acute myeloid leukemia bearing the isocitrate dehydrogenase 1 mutation. Blood.

[20] Ward P.S., Patel J., Wise D.R., Abdel-Wahab O., Bennett B.D., Coller H.A., Cross J.R., Fantin V.R., Hedvat C.V., Perl A.E. (2010). The common feature of leukemia-associated idh1 and idh2 mutations is a neomorphic enzyme activity converting alpha-ketoglutarate to 2-hydroxyglutarate. Cancer Cell.

[21] The Cancer Genome Atlas Research Network (2013). Genomic and epigenomic landscapes of adult de novo acute myeloid leukemia. New Engl. J. Med..

[22] Abbas S., Lugthart S., Kavelaars F.G., Schelen A., Koenders J.E., Zeilemaker A., van Putten W.J., Rijneveld A.W., Lowenberg B., Valk P.J. (2010). Acquired mutations in the genes encoding idh1 and idh2 both are recurrent aberrations in acute myeloid leukemia: Prevalence and prognostic value. Blood.

[23] Marcucci G., Maharry K., Wu Y.Z., Radmacher M.D., Mrozek K., Margeson D., Holland K.B., Whitman S.P., Becker H., Schwind S. (2010). IDH1 and IDH2 gene mutations identify novel molecular subsets within de novo cytogenetically normal acute myeloid leukemia: A cancer and leukemia group b study. J. Clin. Oncol..

[24] Paschka P., Schlenk R.F., Gaidzik V.I., Habdank M., Kronke J., Bullinger L., Spath D., Kayser S., Zucknick M., Gotze K. (2010). IDH1 and IDH2 mutations are frequent genetic alterations in acute myeloid leukemia and confer adverse prognosis in cytogenetically normal acute myeloid leukemia with npm1 mutation without flt3 internal tandem duplication. J. Clin. Oncol..

[25] Zou Y., Zeng Y., Zhang D.F., Zou S.H., Cheng Y.F., Yao Y.G. (2010). IDH1 and IDH2 mutations are frequent in chinese patients with acute myeloid leukemia but rare in other types of hematological disorders. Biochem. Biophys. Res. Commun..

[26] Patel J.P., Gonen M., Figueroa M.E., Fernandez H., Sun Z., Racevskis J., Van Vlierberghe P., Dolgalev I., Thomas S., Aminova O. (2012). Prognostic relevance of integrated genetic profiling in acute myeloid leukemia. New Engl. J. Med..

[27] Yamaguchi S., Iwanaga E., Tokunaga K., Nanri T., Shimomura T., Suzushima H., Mitsuya H., Asou N. (2014). IDH1 and IDH2 mutations confer an adverse effect in patients with acute myeloid leukemia lacking the npm1 mutation. Eur. J. Haematol..

[28] Xu X., Zhao J., Xu Z., Peng B., Huang Q., Arnold E., Ding J. (2004). Structures of human cytosolic nadp-dependent isocitrate dehydrogenase reveal a novel self-regulatory mechanism of activity. J. Biol. Chem..

[29] Emadi A., Jun S.A., Tsukamoto T., Fathi A.T., Minden M.D., Dang C.V. (2014). Inhibition of glutaminase selectively suppresses the growth of primary acute myeloid leukemia cells with idh mutations. Exp. Hematol..

[30] Fathi A.T., Wander S.A., Faramand R., Emadi A. (2015). Biochemical, epigenetic, and metabolic approaches to target idh mutations in acute myeloid leukemia. Semin. Hematol..

[31] Jansen A.J., Caljouw M.A., Hop W.C., van Rhenen D.J., Schipperus M.R. (2004). Feasibility of a restrictive red-cell transfusion policy for patients treated with intensive chemotherapy for acute myeloid leukaemia. Transfus. Med..

[32] Schlaifer D., Cooper M.R., Attal M., Sartor A.O., Trepel J.B., Laurent G., Myers C.E. (1993). Myeloperoxidase: An enzyme involved in intrinsic vincristine resistance in human myeloblastic leukemia. Blood.

[33] McGrath T., Center M.S. (1988). Mechanisms of multidrug resistance in hl60 cells: Evidence that a surface membrane protein distinct from p-glycoprotein contributes to reduced cellular accumulation of drug. Cancer Res..

[34] Ozgen U., Savasan S., Stout M., Buck S., Ravindranath Y. (2000). Further elucidation of mechanism of resistance to vincristine in myeloid cells: Role of hypochlorous acid in degradation of vincristine by myeloperoxidase. Leukemia.

[35] Yang L., Panetta J.C., Cai X., Yang W., Pei D., Cheng C., Kornegay N., Pui C.H., Relling M.V. (2008). Asparaginase may influence dexamethasone pharmacokinetics in acute lymphoblastic leukemia. J. Clin. Oncol..

[36] Kawedia J.D., Liu C., Pei D., Cheng C., Fernandez C.A., Howard S.C., Campana D., Panetta J.C., Bowman W.P., Evans W.E. (2012). Dexamethasone exposure and asparaginase antibodies affect relapse risk in acute lymphoblastic leukemia. Blood.

